# Brand design in the era of 5g new media and its impact on consumers’ emotional experience

**DOI:** 10.3389/fpsyg.2022.956490

**Published:** 2022-09-30

**Authors:** Xinru Li

**Affiliations:** College of Arts, Humanities and Social Sciences, University of Edinburgh, Edinburgh, United Kingdom

**Keywords:** 5g era, new media, brand design, emotional experience, consumers

## Abstract

Brand design is an important part for enterprises to improve brand awareness and attract consumers. If a company wants to develop for a long time, it must have a good brand image. Good brand design can make a deep impression on consumers, thereby promoting purchase intention. With the advancement of technology and the development of the times, traditional brand design can no longer meet the needs of consumers, and the design of brand experience has gradually become a trend. Incorporating emotional experience into brand design can influence consumers’ brand decisions, and can also make consumers resonate with the brand, which in turn generates purchase behavior. New media is a media state produced within the framework of new technologies and technological development systems. In the context of the 5g new media era, this paper studies and analyzes brand design and its impact on consumers’ emotional experience, aiming to change the traditional brand design method and provide new ideas for brand design. In addition, combined with new media technology and mathematical relational model, the brand design method based on consumer emotion is discussed. The results show that the brand design method proposed in this paper can increase the emotional experience of consumers and improve the brand praise by 7.3%.

## Introduction

If an enterprise wants to stand out in the fierce market competition, it must pay attention to the building of its brand image. The brand image of an enterprise is actually the face of an enterprise, and having a good brand image can promote the enterprise to achieve better benefits. A good brand image will attract consumers and achieve the role of independent communication, thereby enhancing consumers’ sense of exclusiveness and recognition of the brand. With the change of people’s life style, the traditional way of brand design can no longer meet the needs of consumers. Emotional marketing starts from the emotional needs of consumers, arouses and arouses the emotional needs of consumers, induces the resonance of consumers’ minds, embodies emotions in marketing, and allows affectionate marketing to win ruthless competition. In the new era, consumers pay more attention to the emotional experience brought by brands, and it has become a trend to add emotional marketing elements to brand design.

With the rapid development of network technology, the 5g new media era has quietly arrived. Shan discussed three modes of reading guide in the new media era, and analyzed the problems existing in the mode of reading guide. Finally, a joint reading guidance system based on new media is established, which provides an important basis for the development of reading guidance (Shan, 2021). Gomez-Barquero proposes a new media teaching evaluation framework, which aims to promote the development of learning models in the new media era. The results show that the evaluation framework can accurately evaluate the teaching quality in the course (Gomez-Barquero et al., 2020). Fu put forward innovative ideas for animation development based on new media, introduced the project teaching method into the studio, strengthened cooperation with enterprises, and explored new cooperation models. His research shows that new media has improved the research ability of animation technology (Fu, 2021). Zhang discussed the dynamics of international communication in the age of new media technologies. The results showed that new media technology can remove barriers of distance and time, reduce the imbalance of news flow, promote international relations, and actively promote globalization (Zhang and Kou, 2021). Li conducted research on rural e-commerce in the era of new media, and found that new media created necessary conditions for the development of rural e-commerce and promoted rural consumption levels and industrial development (Li, 2022). Liu studied and analyzed the impact of new media on the news consumption market, and found that when news consumers choose news content, it is not based on the quality of news content and the functions of news media, but on their own convenience (Liu, 2020). Wang analyzed the visual reading product system from the dual perspectives of visualization and new media, and constructed a new media-based visual reading system. The results found that new media has important reference value for the construction and development of reading systems (Wang, 2021). The research work in these new media eras is relatively detailed, but does not involve the impact of brand design on consumers’ emotions.

Brand design based on consumer emotional experience is one of the important strategies of emotional marketing. Gong and Zuo team explored the impact of gender stereotypes and issue advocacy on consumers’ emotional experience. The results showed that problem advocacy can counteract the negative effects of traditional female stereotypes (Gong and Zuo, 2020). Chiou assessed the impact of simple and complex brand identities on food consumer sentiment. The evaluation results showed that brand logo design affects the actual food intake of food consumers (Chiou and Wang, 2017). Gilal used sentiment analysis method to study the influence of consumer sentiment on new brand design, and finally found that research on consumers’ emotional needs is beneficial to the personalized design of brands (Gilal et al., 2020). Choi proposed a consumer sentiment analysis method based on consumer psychology and behavior theory. Finally, experiments showed that this method was very suitable as an analysis tool for consumer sentiment problems (Choi and Yoon, 2019). Huang constructed a product design evaluation model based on consumer experience, which meet the clear and unique needs of consumers and provides important suggestions for improving product design and better user experience (Huang et al., 2021). The Ren and Xu teams examined the impact of brand experience on the dimension of brand equity from the perspective of consumers. The results showed that brand experience directly affects consumers’ brand satisfaction (Ren and Xu, 2021). Wang proposed a consumer decision-making model based on benefits and risks. The experimental results showed that both perceived benefit and perceived risk had a strong impact on purchase intention (Wang, 2017). The above content specifically analyzes the impact of brand design on consumer sentiment, but does not introduce brand design in the era of 5g new media.

This paper studies brand design based on consumers’ emotional experience in the era of 5g new media, pointed out that the new brand design is not only a formal innovation or a change in the brand marketing strategy, but should aim to create a new brand visual image and enhance the emotional experience of consumers. Only in this way can we continue to help companies develop design solutions that conform to the trend of the new era. Only in this way can we continuously help enterprises to cultivate design schemes and mechanisms that cater to the new era, so that the corporate culture can be better sublimated. Finally, through the cultural connotation and emotional elements in the brand image design, we can improve a perfect purchasing experience for consumers.

## 5g new media era

**FIGURE 1:**
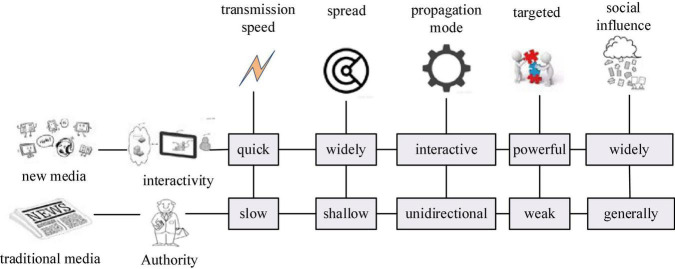
Comparison of new media and traditional media.

New media is a form of dissemination that uses digital technology to provide users with information and services through channels such as computer networks, wireless communication networks, satellites, and terminals such as computers, mobile phones, and digital televisions. There is no unified definition of what new media is, such as mobile terminals, computer networks, blogs, and online videos. Compared with the four traditional media of television, newspapers, radio, and magazines, new media is usually called the fifth media (Muller and Klerk, 2020). The newness of new media refers to innovation, and the newness is in the elements of production technology level, dissemination form, and business philosophy. Figure 1 is a comparative analysis of new media and traditional media. New media has the characteristics of interactivity, immediacy, diversity of content forms, pertinence, and breadth. The Internet is the main carrier and form of new media. Compared with traditional media, the interactivity of the Internet is the biggest feature of new media. As a new means of dissemination, new media can rely on network technology to continuously change the way of information dissemination. New media itself, as a communication medium, constantly uses new technologies to change the way of information transmission. It is not only a carrier that carries content, but also carries the emotions and preferences, needs and resonance, personality and even social circles of target users.

With the acceleration of the times, all aspects of work, life, etc. are showing a trend of fragmentation. It is in this trend that new media emerge and develop. People’s information needs are also changing with the innovation of lifestyle, and new media can just meet these changing needs (Benzarti, 2021). The new media marked by the Internet has also entered a stage of personality expression and new communication habits. Human beings are no longer passive information receivers who only read newspapers, and information consumers are also producers at the same time. They need to comment in real time, express their opinions, and participate in online discussions, so interactive information dissemination methods become particularly important.

## Brand design in the new media era

### Brand name

Brand names must reflect the brand’s values or the positioning of the product. The most commonly used naming methods in the new media age are: naming animals or objects; naming with the initials of people’s names; naming with the names of places, etc. The naming should follow the principles of Lenovo’s active, innovative content and unique creativity. Then, it is necessary to combine the communication attributes of new media to give the brand a strong communication force, especially to make a deep impression on the established goals of the brand. Brand names should be strong, clear, and short, preferably 2–4 words. The audience of new media is mostly young people. The characteristics of these people are fashion and simplicity. It is difficult for them to recognize the names that are too complicated.

### Brand type design

**FIGURE 2:**
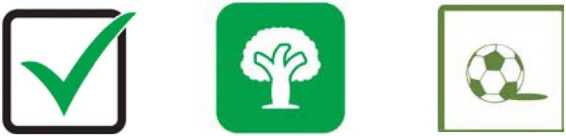
Square class icon.

The brand type of an enterprise includes branding decision-making, brand mode selection, brand identification definition, brand extension planning, and brand management planning. The design of brand types can be basically divided into two categories. The first category is square and rounded square. As shown in Figure 2, such brand logos are common, and most of the logos in the APP brand logo can be classified into this category.

### Brand design content in the new media era

Brand communication refers to various direct and indirect methods, by which companies inform consumers about brand information, persuade consumers to buy brands, and maintain brand memory. Nowadays, enterprises not only need static visual brand communication, but also need to adapt to the brand design under the new media. The content of traditional brand design is mainly to design related plane things, but this can no longer meet the needs of brand design in the new era. The traditional brand design method must make changes to keep up with the changes of the times in order to adapt to the current trend, which is also a necessary condition for brand design in the new media era (Wanrudee and Xiaobing, 2018). In the context of the rapid development of the new media era, it is difficult for brand design to rely on graphic printing to meet the needs of consumers in modern society for the richness, immediacy and infinity of brand information dissemination, the way of brand design must be changed and innovated.

## The influence of brand design on consumers’ emotional experience

**FIGURE 3:**
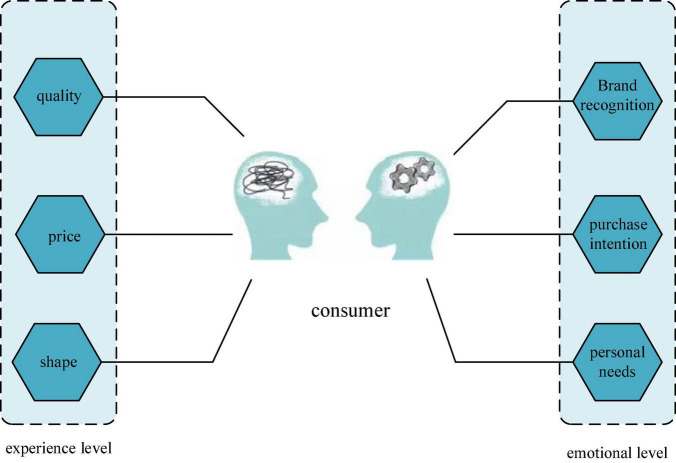
Consumer brand experience.

Consumer behavior research shows that brand experiences occur when consumers search for products, purchase products, and use products and services. As shown in Figure 3, brand experience is the internal thinking (perception, emotion) and behavioral responses expressed by consumers through related factors (product identification, packaging design, product communication) when they contact the product. The experience process can be divided into functional and emotional levels. The functional level includes consumers’ experience of product price, quality and appearance. The emotional level includes consumers’ thinking on purchase intention, personal needs, and brand perception.

**FIGURE 4:**
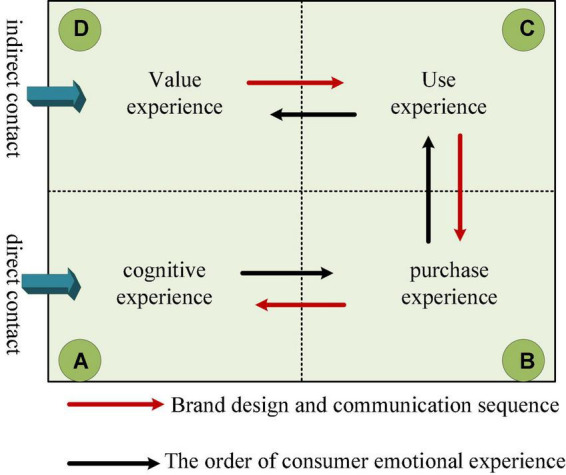
The relationship between consumer experience and brand design.

The design type of brand experience is related to product service and consumer experience, and consumers have product experience when they come into contact with the product. When consumers are in physical contact with the product, it is a direct product experience; when the product is displayed through advertising, it is an indirect product experience. These consumer experiences are multi-dimensional, such as fun, emotion, and imagination (Ijabadeniyi and Govender, 2019). The brand experience varies in intensity and length, and it also has different valences. Consumers may experience positive or negative, short-lived or long-lasting experiences. A lasting brand experience stored in consumers’ memories affects consumer loyalty and satisfaction. Emotional experience refers to an individual’s awareness of one’s own emotional state. Consumer emotional experience includes indirect contact and direct contact, both of which are consumers’ attitude experience on whether the brand meets their needs (Zheng et al., 2022). Figure 4 shows the relationship between consumer experience and brand design. When building a brand image, only the rigid needs of enterprises are often considered, while the most important emotional needs of consumers are ignored. In this way, the influence of the brand is difficult to combine with the needs of the public, and the value of the brand is difficult to see. Emotional identification is the emotional response and high affirmation of the consumer group after the brand’s empirical activities. The degree of emotional identity varies from person to person and from brand to brand, and the level of recognition will directly or indirectly affect the development of the brand.

The main goal of brand image design in the media age is to obtain consumers’ high emotional recognition of the brand. The main reasons are as follows: First, when the company builds its brand image, it does not take into account the cultural and emotional needs of the brand itself or the emotional needs of the public, and the brand is not recognized by the public, let alone emotional recognition. Secondly, in the company’s brand image design activities, if the brand concept and the needs of the public’s emotional experience are not fully considered, it will be difficult to attract the public to experience your brand, and it will be even more difficult to achieve a high level of emotional recognition. Then, brand image building activities take into account the needs of all parties but lack novel designs, making it difficult for people to have a good experience. Although emotional identity can be formed, the degree of emotional identity is not high. Finally, brand awareness and credibility directly affect the public’s emotional perception of the brand. If there are negative factors in a brand company, it will greatly affect the public’s perception of the brand’s popularity and credibility, and it is difficult to have a good impression of the company.

## Brand color design methods and the mathematical relationship between brands and consumers

### Color design

Fixed weights are weights that are assigned fixed values to component bonds according to certain rules. Usually, fixed weights mainly include equal weights, hierarchical equal weights, optimized equal weights, and other fixed weights. The most commonly used color scheme is the fixed-weight grayscale image-to-color image algorithm (Özbeyaz, 2021). In the field of grayscale image to color image conversion, some classical algorithms combine three grayscale image channels into color images according to a constant weight. The advantage of this is that the computing power is very small, it can process image files in large batches, and in many common cases, it can also deal with the need for coloring grayscale images. The component method is to process the pixel value of one of the three RGB elements in the grayscale RGB image into the color luminance value of the color image to generate a color image. Various items can be selected according to the actual application, expressed as Formula (1). The converted color image is denoted as *f*(*i*, *j*), and *R*(*i*, *j*), *G*(*i*, *j*), and *B*(*i*, *j*) are the three components of RGB in the color image, respectively.


(1) f⁢(i,j)=R⁢(i,j) o⁢r f⁢(i,j)=G⁢(i,j) o⁢r f⁢(i,j)=B⁢(i,j)

The maximum method is to subtract the maximum value of three RGB pixels from each RGB image in grayscale as the color of the color image. The expression is Formula (2).


(2) f⁢(i,j)=max⁡(R⁢(i,j),G⁢(i,j),B⁢(i,j))

The minimum method is to derive the minimum value of the three RGB elements of each pixel of the RGB grayscale image as the color of the color image. The expression is as Formula (3).


(3) f⁢(i,j)=min⁡(R⁢(i,j),G⁢(i,j),B⁢(i,j))

The average method is to take the average value of the three RGB components of the grayscale image as the grayscale value of the color image. The expression is as Formula (4).


(4) $$f\,\left(i,j\right)=\frac{R\,\left(i,j\right)+G\,\left(i,j\right)+B\,\left(i,j\right)}{3}$$

The weighted average method is to use the result obtained by calculating the three RGB elements of the grayscale image according to a certain weight as the color value of the color image, such as Formula (5).


(5) $$\left\{ \begin{array}{c} f\,\left(i,j\right)=w_{r}\,R\,\left(i,j\right)+w_{g}\,G\,\left(i,j\right)+w_{b}\,B\,\left(i,j\right)\\ w_{r}+w_{g}+w_{b}=1\\ w_{r}\geq 0,w_{g}\geq 0,w_{b}\geq 0 \end{array}\right.$$

Among them, *w*_*r*_, *w*_*g*_, *w*_*b*_ are the weights of the three RGB elements. Experiments show that when the weight values of the three RGB elements are *w*_*r*_ = 0.2989, *w*_*g*_ = 0.5870, and *w*_*b*_ = 0.1140, respectively, the generated color image has an ideal effect, and its adaptability to different images is also better than the first three (Manchaiah et al., 2021). This method is also the element Y in the NTSC Rec. 601 standard, and Matlab also includes it in the function, also known as the Luminance method, and the expression is as Formula (6).


(6) f⁢(i,j)=0.2989*R⁢(i,j)+0.5870*G⁢(i,j)+0.1140*B⁢(i,j)

The fixed weight algorithm is a relatively simple and convenient algorithm in the field of gray image colorization. Colorizing grayscale images is a process of converting three-dimensional image data into one-dimensional color and brightness data, and the information loss in this process is very large. Therefore, many researchers try to preserve the original image information in the process of colorizing grayscale images, but the fixed-weight algorithm still has many shortcomings in preserving the original image information.

### Mathematical relationship between product brands and consumers

Brand utility reflects the degree of satisfaction of people’s needs in the process of consuming something or being served. Consumers acquire the use value of commodities by purchasing commodities, thus realizing the demand for a desire, and always maximize a desire under their existing conditions, that is, the maximization of utility. The use value is closely related to the utility of the product, and the use value is the ratio of the quality of the product to the price of the product, that is, use value (f) = quality (m)/price (p). The theory of the model assumes that consumers only buy one product of a certain type, the price of this product is P, the quantity is q, its disposable income is I, and the surplus after purchasing this product is s. The consumer’s income constraint is:


(7) p⁢q+s=I

The consumer’s utility function is:


(8) U=U⁢(f,q,s)

The mathematical model of consumers’ pursuit of utility maximization is as follows:


(9) max⁡U=(f,f⁢q,s)

(10) s.t⁢p⁢q+s=I

Removing the constraints and substitute into the utility function to get: *max*⁡*u*(*f*,*q*,*s*) = *u*(*f*,*q*,1−*pq*). According to the Cobb-Douglas utility number (Perrea et al., 2017), choosing an appropriate coordinate system, the utility function can be written in the following form:


(11) $$\begin{array}{c} u\,\left(f,q,I-p\,q\right)=A\,f^{\alpha }\,q^{\beta }+k\,\left(I-p\,q\right)\\ \left(A>0,k>0,0<\alpha <y,0<\beta <1\right) \end{array}$$

Although the quality, price and quantity of similar products available in the market are discrete, once consumers decide to buy a certain product, its use value has been selected. For consumers to maximize their utility, the quantity of the product must satisfy:


(12) $$\frac{\sigma \,u}{\sigma \,q}=0$$

That is


(13) $$A\,\beta \,f^{\alpha }\,q^{\beta -1}-k\,p=0$$

From Formula (4), the optimal purchase quantity can be obtained as:


(14) $$q={\left(\frac{\beta \,A}{k\,p}\right)}^{\frac{1}{1-\beta }}\,f^{\frac{\alpha }{1-\beta }}$$

Consumer spending on this product is:


(15) $$p\,q={\left(\frac{\beta \,A}{k}\right)}^{\frac{1}{1-\beta }}\,f^{\frac{\alpha }{1-\beta }}\,p^{\frac{\alpha }{1-\beta }}$$

Substituting Formula (15) into Formula (13) to get:


(16) $$u\,\left(f,q,I-p\,q\right)=A\,\left(1-\beta \right)*{\left(\frac{\beta \,A}{k}\right)}^{\frac{1}{1-\beta }}\,f^{\frac{\alpha }{1-\beta }}\,p^{\frac{\alpha }{1-\beta }}+k\,I$$

When the income is constant, it can be obtained by Formula (16):


(17) $$-\frac{\sigma \,u}{\sigma \,q}=A\,\alpha \,{\left(\frac{\beta \,A}{k}\right)}^{\frac{\beta }{1-\beta }}\,f^{\frac{\alpha +\beta -1}{1-\beta }}\,p^{\frac{\alpha }{1-\beta }}$$

Since


(18) $$f=\frac{m}{p},\frac{\sigma \,f}{\sigma \,m}=1$$

So


(19) $$\frac{\sigma \,u}{\sigma \,m}=\frac{\sigma \,u}{\sigma \,f}*\frac{\sigma \,f}{\sigma \,m}=A\,\sigma \,{\left(\frac{\beta \,A}{k}\right)}^{\frac{\beta }{1-\beta }}\,f^{\frac{\alpha +\beta -1}{1-\beta }}\,p^{\frac{\alpha }{1-\beta }}>0$$

The same can be obtained:


(20) $$\frac{\sigma \,u}{\sigma \,p}=\frac{\sigma \,u}{\sigma \,f}*\frac{\sigma \,f}{\sigma \,p}+\frac{\sigma \,u}{\sigma \,p}=-A\,{\left(\frac{\beta -1}{k}\right)}^{\frac{\beta }{1-\beta }}*\left(\alpha f^{\frac{\alpha +\beta -1}{1-\beta }}p^{\frac{\alpha }{1-\beta }}+\beta f^{\frac{\alpha +\beta -1}{1-\beta }}p^{\frac{\alpha }{1-\beta }}\right)<0$$

It can be obtained from the above formula that when the consumer’s income is constant, the consumer’s utility function is positively correlated with the quality of the purchased goods. This also proves that the quality of the product and the popularity of the product brand have always been a necessary condition for consumers’ purchasing needs.

## Results of brand design and consumer emotional experience in the new media era

Through the questionnaire to understand consumers’ views on the use of new media in brand design, consumers’ identification and emotional needs for brand design under new media are obtained. 100 consumers of different age groups were sampled to analyze their influence by brands and the frequency of purchasing branded clothing.

**FIGURE 5:**
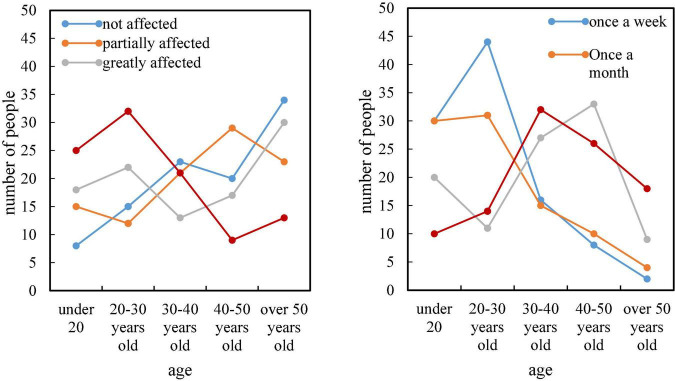
Consumers of different age groups are influenced by brands and purchase branded clothing frequency.

As can be seen from Figure 5, only a small number of respondents are completely unaffected by the brand when purchasing clothing, while other consumers are more or less affected by the brand, and nearly half of consumers buy branded clothing more frequently. It can be seen from this that not only clothing companies are aware of the importance of brands, but more and more consumers are becoming more and more aware of brands.

**FIGURE 6:**
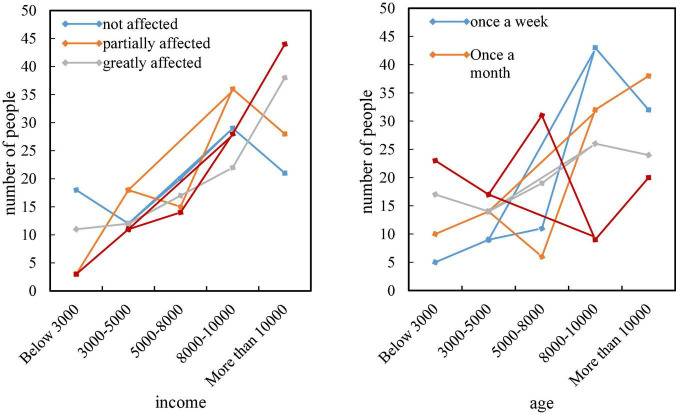
Consumers with different incomes are influenced by brands and how often they buy branded clothing.

Figure 6 shows the degree of brand influence and the frequency of purchasing branded clothing for 100 consumers with different incomes. It can be seen from the line chart that with the continuous increase of income, the degree of brand influence is getting higher and higher, and the frequency of purchasing brand clothing is relatively high. But there are exceptions, the influence degree and purchase frequency of consumers with an income of more than 10,000 will occasionally decline. This part of the people may pursue higher-level things, and have become numb to the brand.

**FIGURE 7:**
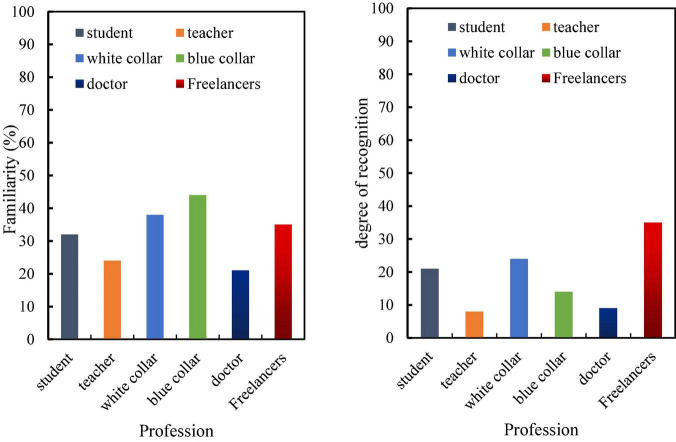
Familiarity and awareness of different consumers with new media brand design.

Figure 7 shows the level of understanding of different consumers in the survey about brand design in the new era, types of consumers such as students, teachers, and doctors have a lower level of awareness of brand design, while white-collar, blue-collar, and freelancers have a higher level of awareness of brands. In terms of recognition, consumers with different education levels have different recognition of new brands, and the brand recognition of freelancers is the highest.

Taking into account the brand value and customers’ purchasing thoughts as a measure of brand loyalty, a benchmark scale was used for data analysis. Table 1 shows the exploratory factor analysis of the customer’s brand emotional experience scale.

**TABLE 1:** Analysis of brand emotional experience factors.

Construct	Project	Emotion	Behavior	Reliability
Brand emotional experience	Does brand affect emotions?	0.689		0.647
	Have an emotion for the brand	0.708		
	Is an emotional brand	0.739		
	Brand experience		0.813	0.718
	Physical activity while using the brand		0.817	

Brand experience results were analyzed using structural equation modeling.

It can be seen from Table 2 that consumers’ perception of brands also affects the relationship structure between brands and consumers. A consumer who likes a high-emotion brand experience perceives a brand as trustworthy. That is, when consumers feel brand emotion, their relationship with the brand strengthens and they begin to trust the brand. At the same time, when consumers acquire high-quality emotional and behavioral quality feelings, the level of quality commitment will also be greatly improved. Consumer perceptions of brands also affect the relationship structure between brands and consumers.

**TABLE 2:** Structural equation test results.

Assumption	Path	Regression value	*P*-value	Result
X1a	Emotional brand experience→brand trust	0.743	0.001	Support
X1b	Behavioral brand experience→brand trust	−0.612	0.175	Support
X1c	Emotional brand experience→brand promise	0.246	0.018	Support
X1d	Behavioral brand experience→brand promise	0.158	0.013	Support
X2	Brand trust→brand promise	0.403	0.002	Support

**FIGURE 8:**
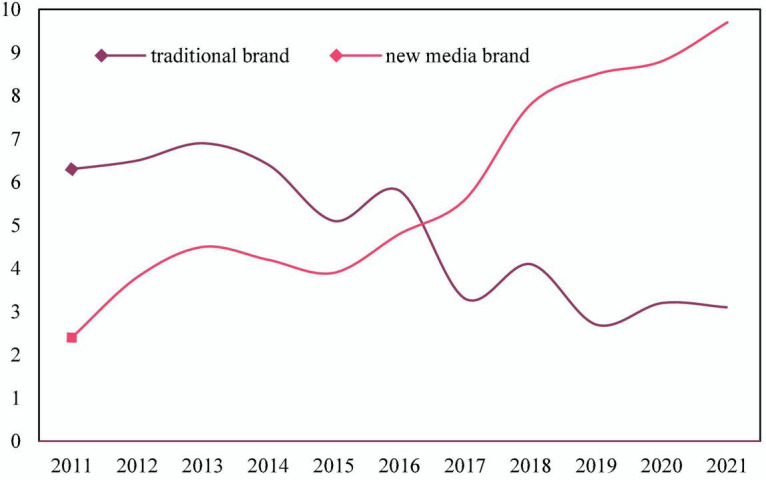
Consumers’ praise for traditional and new-age brands from 2011 to 2021.

Finally, we compare consumers’ favorable comments on traditional brands and new-age brands from 2011 to 2021, as shown in Figure 8. Since 2011, the popularity of traditional brands has been in a high position. But over time, the ratings dropped significantly by 2015, and fluctuated until 2021, but the trend has been declining. The positive ratings of new-age brands were at a low level in 2011, but they have been on the rise over time. Compared with traditional brands, new media brands have a 7.3% higher overall rating.

## Conclusion

As a form of new media, 5g new media not only creates a new design environment for brand building, but also reveals new design needs. Enterprises should accelerate brand recognition, use brand design to convey emotional tone, touch consumers’ spiritual needs, arouse their emotional resonance, and then deepen consumers’ interpretation of the brand. In today’s increasingly fierce market competition, cost performance and service are no longer the only competitiveness of brand enterprises, and consumers pay more attention to the emotional value of the brand. Paying attention to the cultural level of the brand in the process of corporate brand design, so that consumers can experience the affiliated emotions from the brand, is an important strategy for brand design in the new 5g era.

## Data availability statement

The original contributions presented in this study are included in the article/supplementary material, further inquiries can be directed to the corresponding author.

## Author contributions

XL: conception and writing of the manuscript, data investigation, and approved the submitted version.
